# Trophic ecology of two co-existing Sub-Antarctic limpets of the genus *Nacella*: spatio-temporal variation in food availability and diet composition of *Nacella magellanica* and *N. deaurata*

**DOI:** 10.3897/zookeys.738.21175

**Published:** 2018-02-19

**Authors:** Sebastián Rosenfeld, Johanna Marambio, Jaime Ojeda, Juan Pablo Rodríguez, Claudio González-Wevar, Karin Gerard, Tamara Contador, Gemita Pizarro, Andrés Mansilla

**Affiliations:** 1 Laboratorio de Ecosistemas Marinos Antárticos y Subantárticos, Universidad de Magallanes, Casilla 113-D, Punta Arenas, Chile; 2 Instituto de Ecología y Biodiversidad (IEB) Casilla 653, Santiago, Chile; 3 GAIA Antártica – Universidad de Magallanes, Departamento de Recursos Naturales, Bulnes 01890, Punta Arenas, Chile; 4 Parque Etnobotánico Omora, Universidad de Magallanes, Teniente Muñoz 396, Puerto Williams, Chile; 5 Instituto de Fomento Pesquero, Casilla 101, Punta Arenas, Chile

**Keywords:** Gastropoda, Magellanic Province, herbivory, macroalgae, Nacellidae, periphyton

## Abstract

Interactions between algae and herbivores can be affected by various factors, such as seasonality and habitat structure. Among herbivores inhabiting marine systems, species of the order Patellogastropoda are considered key organisms in many rocky coasts of the world. *Nacella* species are one of the most dominant macro-herbivores on the rocky shores of the sub-Antarctic ecoregion of Magellan. However, the importance of its key role must be associated with its trophic ecology. The objective of this work was to evaluate spatial and temporal variabilities in the dietary composition of two intertidal *Nacella* species, considering grazing on macro- (macroalgae) and microscopic (periphyton) food. The composition of periphyton and the availability of macroalgae in the winter and summer seasons were examined at two localities of the Magellanic province, alongside the gut contents of *N. magellanica* and *N. deaurata*. The dietary composition differed between the two *Nacella* species, as well as between seasons and locations. The differences observed in the diet of the two species of *Nacella* may be mainly due to their respective distributions in the intertidal zone. Both species presented a generalist strategy of grazing, which is relationed to the seasonality of micro- and macroalgae availability and to the variability of the assemblages between the localities. This research was the first to perform a detailed study of the diet of intertidal *Nacella* species.

## Introduction

The structure and dynamics of intertidal ecosystems depend on both abiotic factors (e.g., temperature, substrate, and climate) and biological interactions (e.g., predation, competition, recruitment) (Castilla and Dúran 1985, Menge 2000, Díaz and McQuaid 2011, Murray et al. 2016). Among the biological interactions, predation through herbivory is one of the most relevant processes, since it helps determine the structure and functioning of ecosystems (Camus et al. 2008). In rocky shores ecosystems, herbivory can modify the spatial and seasonal patterns of algal communities (Aguilera 2011). These changes are not only related to the presence or abundance of grazers but also to the species to which it belongs (species identity) (O'Connor and Crowe 2005). However, interactions between algae and the different species of grazers can be directly or indirectly affected by multiple factors (Iken 1999). Among these factors, seasonality affects the coverage and biomass of micro- and macroalgae (Cubit 1984, Santelices 1987, Iken 1999, Jenkins et al. 2001), and consequently the diet of intertidal herbivorous species (Hill and Hawkin 1991). Seasonality itself may vary depending on the latitudinal range of rocky coasts, since, seasonal fluctuations in biomass of algae communities (micro and macroalgae) over the year increase with the latitude (Hill and Hawkin 1991, Thompson et al. 2000, Jenkins et al. 2001, Ojeda 2013), affecting the availability of food for different species of the food chain, including herbivores (Hill and Hawkin 1991). At the same time, habitat structure and high environmental heterogeneity imply spatial variation in the richness, abundance and structure of algae communities (Hill and Hawkin 1991, Benedetti-Cecchi and Cinelli 1997, Ojeda 2013), thus affecting the diet of herbivores (Branch 1971).

The main investigations on the ecology of herbivore-algae interactions have been carried out on molluscs of the order Patellogastropoda (Rubio et al. 2015). Patellogastropods or ‘true limpets’ are some of the most ubiquitous mollusks composing the marine littoral hard-substrate communities (Ponder and Lindberg 1997). Most of the ‘true limpets’ are herbivorous grazers, some on macroalgae, but a majority on sessile aquatic biota, attached to submerged substrate including diatoms, algal spores, detrital deposits and other bottom organism in combination with microbial bio-films (periphyton) (Branch 1981, van Dam et al. 2002). Much of the herbivore-algae interactions have been studied from an experimental point of view, evaluating the effect of exclusion or predation on the colonization of macroalgae and invertebrates (Hill and Hawkin 1991, Díaz and McQuaid 2011, Rubio et al. 2015). Nevertheless, research concerning gut contents is not so extensive within patellogastropod limpets (see Rubio et al. 2015). This information will highlight the roles of species in the functioning of coastal systems (Camus et al. 2008, Aguilera 2011).

Following this, the current paradigm of herbivory in “limpets” has recently changed (Rubio et al. 2015). For example, studies have addressed the role of animal items in some patellogastropod species (Camus et al. 2008), indicating that these limpets actually present omnivorous habits, thus affecting the classical trophic networks in intertidal systems (Camus et al. 2008, Rubio et al. 2015). Small-scale studies of trophic ecology in patellogastropods have highlighted the dietary variability among congeneric species mainly due to their spatial distribution (Santina et al. 1993). Branch (1971), from the study of the trophic ecology of 11 patellogastropods species along the South-African coast, found that the environmental heterogeneity between sites (e.g., different types of substrates, coastal geomorphology, wave exposure) is related to the variability of algal communities, which influences the species diet. Meanwhile, on a seasonal scale, patellogastropods demonstrated a generalist diet that follows the available food in the habitat (Branch 1971, Hill and Hawkin 1991, Jenkins et al. 2001, Aguilera 2011). It is important to note that among patellogastropods with generalist habits, some species are exclusive consumers of periphyton (not macroalgae) (Chapman and Underwood 1992). Therefore, the diet and food preferences of key herbivores like patellogastropods and their temporal and spatial dynamics will help us to better understand the functioning of the rocky coastal environment.

The rocky shores of the channels and fjords of the Magellan Ecoregion present high environmental heterogeneity, influenced by several geomorphological (e.g., type of substrate; Ojeda et al. 2014), oceanographic (e.g., salinity variation; Silva and Calvete 2002), climate (e. g. seasonal variation in solar radiation and temperature; Marambio et al. 2017) and biological (e.g., diversity of biotopes associated with macroalgae; Soto et al. 2012) factors. The richness of patellogastropods in the Magellanic Province is represented by eleven species (Linse 1999), among which the most represented genus is *Nacella* (Valdovinos and Rüth 2005, Aranzamendi et al. 2009, González-Wevar et al. 2010, González-Wevar et al. 2011), a dominant group among the macro-benthic invertebrates, especially on rocky shores (Guzmán 1978, Ojeda et al. 2014). Along the Strait of Magellan and the sub-Antarctic channels and fjords this group also has an important role in the feeding habits of local communities (indigenous people, fishermen; Ojeda 2013). According to lastest phylogenetic studies of the group, four valid taxonomic units are recognized in the Magellanic Province: *Nacella magellanica* (Gmelin, 1791), *N. deaurata* (Gmelin, 1791), *N. flammea* (Gmelin, 1791) and *N. mytilina* (Helbling, 1779). Despite the dominance of the four species on the coastal zone, some differences in the distribution are visible; i) only *N. deaurata* and *N. magellanica* live in rocky intertidal environments (Aranzamendi et al. 2009, González-Wevar et al. 2011), ii) *N. magellanica* is more common in the middle intertidal zone, and iii) *N. deaurata* distributes from the middle and lower intertidal zone to the shallow subtidal (González-Wevar et al. 2011, Rosenfeld 2016). However, related information about the trophic ecology of *Nacella* species is still under study. The diet of *Nacella* intertidal species (Andrade and Brey 2014, Andrade et al. 2016) was evaluated upon gut content and stable isotopes but only at a very local level (one time and locality) and evaluating macroscopic food content, however the complete characterization of the diet (microalgae, macroalgae and invertebrates) and the temporal and spatial variations have not yet been addressed. Therefore, the dynamics of the trophic ecology of *Nacella* species remains poorly understood and is essential to comprehend the functioning of the rocky shores ecosystem of the Magellanic channels and fjords. Our objective was to determine the dietary composition of the intertidal species *N. deaurata* and *N. magellanica* across spatial and temporal variations, and evaluate the differences between the two species.

## Material and methods

**Figure 1.**
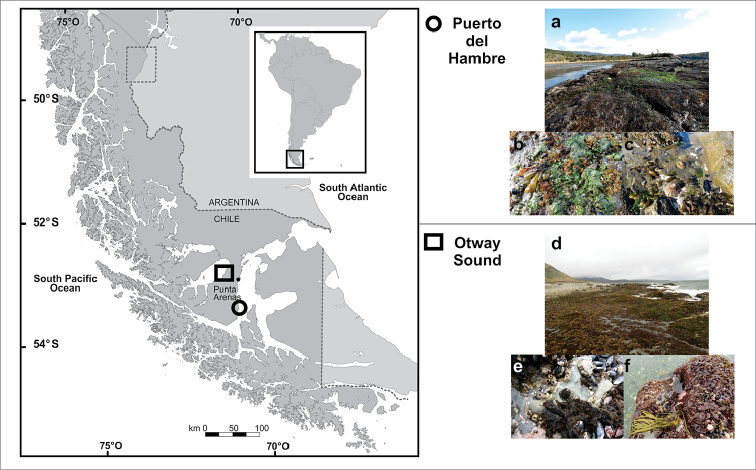
Location of study sites, circle = Puerto del Hambre and square = Otway Sound. Abbreviations: **a, d** general view of both localities **b, e** images of the middle intertidal **c, f** images of the lower intertidal.

The present study was carried out in two locations of the Magellanic Province: Puerto del Hambre (53°37'S, 70°54'W), Strait of Magellan and Otway Sound (53°04'S, 71°19'W; Fig. 1). Puerto del Hambre is in the central microbasin of the Strait of Magellan (Valdenegro and Silva 2003), the central microbasin, is filled with Estuarine Water, that is the result of the mixture of Subantarctic Waters from the Pacific with fresh water, coming from the western microbasin, which move towards the east overflowing the Charles III island constriction and sinking into the central basin and filling it, because of its higher density. The entire central micro-basin column is composed of saline estuarine water and average salinity values of 30.8 psu and average temperature of 7 °C (Valdenegro and Silva 2003). The substrate is characterized by intertidal plataforms, constituted by large rocky extensions regularly covered during the tide (Pisano 1980). Otway Sound is approximately 151 km long from west to east and is a post-glacial lake (Kilian et al. 2007). Geographically, it presents a connection to the Skyring Sound through the Fitz Roy Channel and to the western side of the Strait of Magellan through the Jerónimo Channel (Valdenegro and Silva 2003). Otway Sound presents less saline superficial water (27.75 psu) that flows into the Strait of Magellan through the Jerónimo Channel (Valdenegro and Silva 2003). The substrate is also made of marine plataforms, similar to those of Puerto del Hambre (Pisano 1980).

### Composition and abundance of macroalgae

In order to estimate seasonal variability of algal communities, quantitative sampling of macroalgae was carried out three times throughout the winter season in 2014 (June, July and August) and three times in summer in 2015 (Januar, Februar and March). The NaGISA (Natural Geography in Shores Areas) methodology was used for the quantitative sampling at both study sites, so that the biodiversity of coastal communities was quantified (Iken and Konar 2003). The design of NaGISA sampling protocol is intentionally basic and intends to yield baseline data for the sampling sites (Iken and Konar 2003). Three intertidal levels (high, middle and low according to the vertical height of the shore were assigned to categories defined by Benedetti-Cecchi and Cinelli’s protocol (1997). Sampling was carried out in the two intertidal levels inhabited by *Nacella* species, at middle and lower levels. At each intertidal level, three random quadrats (n = 6) of 50 × 50 cm (area of 2500 cm^2^) were analysed per site. Therefore, the sampling design was of factorial type: 6 (Month) × 2 (localities) × 2 (levels) × 3 quadrats: a total of 72 samples. Each macroalgal sample was placed in a plastic bag for further taxonomic identification (Skottsberg 1941, Wiencke and Clayton 2002, Boraso de Zaixso 2004) at the laboratory. The abundance of macroalgae was determined by dry biomass (g. species^-1^.quadrant^-1^ in 2500 cm^2^) after approximately 48 h at 60 °C (Rutten 2007) and weighed with an analytical balance RADWAG AS 220/C/2, (± 0.0001 g).

### Composition and richness of periphyton

In parallel, the periphyton was sampled following the protocol of Ocaña and Fa (2003). Using a small quadrant of 5 × 5 cm, a hammer and a chisel, a small area of the rock was extracted. Subsequently, the entire surface of the rock was scraped with a brush and preserved in 10% formalin. To estimate the relative abundance of periphyton in the quadrants, a cross-linked Petri dish with 50 points was used to record the number of points of intersection of each taxon, following the protocol of Aguilera et al. (2013). Periphyton sampling was performed at the same intertidal levels and sites as for the macroalgae.

### Gut contents

In order to evaluate the diet, 10 individuals of each *Nacella magellanica* and *N. deaurata* species were collected at each localities from the middle and lower areas of the intertidal levels (Fig. 1a, b, c, d, e, f). In order to determine whether *Nacella* species have a maximum algal availability in summer compared to winter, this sampling was performed three times during summer and three times during winter. Therefore, the sample design was of factorial type: 6 (Month) × 2 (localities) × 2 (species) × 10 individuals = 240 samples. Each individual collected was injected with 10% formaldehyde to preserve the gut contents and allow the subsequent identification of dietary items. To identify the periphyton composition, 1 ml of gut content was analyzed under an inverted microscope with light contrast (Aguilera et al. 2013). A stereomicroscope was used to analyze the macroscopic gut content. The identification of each dietary item was carried out at the most acurrate taxonomic level possible (genus level and species when it was possible). Organisms for which identification is complex, such as diatoms and some specific macroalgae or invertebrate taxa, were only identified at genus level (Aguilera et al. 2013). In the case of macroscopic items, two classifications were made: i) specific level (e.g., species, genus or family) and ii) functional level. The macroalgae were classified upon the structural hardness of the thallus according to Steneck and Watling (1982) and Santelices et al. (2009).

In the gut content analysis, we used the dietary richness (number of dietary items) of each individual of both *Nacella* species, and we calculated the occurrence frequency (%) of each item, which is the proportion of individuals containing each recorded item. In addition, the relative abundance (%) of each item in the digestive tract was estimated for each individual collected. The relative abundance of periphyton in the gastric contents was estimated using an inverted microscope and a reticulated glass slide with 50 points, thus recording the number of points of intersection of each taxon, as in Aguilera et al. (2013). The relative abundance (percentage value) of the macroscopic content was estimated using a stereomicroscope and a plate with a grid of 30 points of intersection, according to Aguilera (2005).

### Data analysis

The composition and abundance of micro- and macroalgae in relation to sampling event, species and height on the shore were determined using univariate and multivariate analyses of biodiversity implemented in the program PRIMER 5.0 (Clarke and Warwick 2001). The univariate variables were: a) species richness S (total number of species identified), b) macroalgal abundance N (dry biomass g species^-1^ quadrant^-1^ in 2500 cm^2^), c) periphyton relative abundance (N% per sample). The univariate (S, N and N %) and multivariate analysis were analyzed with PERMANOVA statistics. Prior to any analyses, the PERMDISP test was performed to assess homogeneity within and between groups (Anderson 2005). The Euclidean distance between pairs of observations was calculated for the univariate analysis (Claudet et al. 2006). For the multivariate variables, Bray-Curtis dissimilarity was calculated between pairs of observations and the data were transformed to the fourth root. All analyses of PERMANOVA were performed with the FORTRAN program (Anderson 2005). The dietary composition of the different *Nacella* species was evaluated calculating the specific importance of each taxa of periphyton, macroalgae and invertebrates per time (winter months or summer months), locality and species, using the multivariate analysis (SIMPER) (Clarke 1993). In addition, we used an MDS analysis (*non-metric multidimensional scaling*; Kruskal and Wish 1978) using Bray-Curtis similarity matrices in order to compare the assemblages of dietary items among the different species and explore the pattern of spatial ordering.

## Results

### Habitat characterization

A total of 17 microalgae (Suppl. material 1: Table S1) and 63 macroalgae (Suppl. material 1: Table S1) taxa were identified in both localities. Statistical analysis showed that the composition of periphyton changes significantly between intertidal levels, between time and between localities (Suppl. material 1: Table S2, S3). In terms of richness and biomass of macroalgae, the highest values were recorded in the sampling at summer in each localities.The macroalgae assemblages in the middle intertidal zone showed a significant increase of richness and average biomass in summer (Suppl. material 1: Table S4, S5). In addition, the PERMANOVA analysis showed that the composition of macroalgae and periphyton varied significantly between levels (middle and lower), between localities and between the winter and summer months (Suppl. material 1: Table S2, S3, S6, S7).

### Diet analyses

Throughout the study period, the periphyton assemblages found in the gut contents were composed of 27 taxa, among which were diatoms, cyanophytes and dinoflagellates (Table 1). Among the identified genera, the most common were *Chroococcus*, *Navicula*, *Pinnularia*, *Cocconeis*, *Fragilaria* and *Licmophora*. It should be noted that the dinoflagellates *Dinophysis*, *Protoperidinium* and *Prorocentrum* were registered in the gut contents of *N. deaurata* and *N. magellanica* in summer at Otway Sound. From a spatial point of view, both *Nacella* species showed significant changes in the dietary composition of periphyton between localities in each time (Suppl. material 1: Table S8, S9).

**Table 1.** Systematic list of items found in the gut contents of *Nacella* species in the winter and summer months, in Puerto del Hambre and Otway Sound, indicating their presence (+).

TAXA	P. Hambre				O. Sound			
	*N. deaurata*		*N. magellanica*		*N. deaurata*		*N. magellanica*	
	Winter	Summer	Winter	Summer	Winter	Summer	Winter	Summer
CYANOBACTERIA								
*Chroococcus* sp.	+	+	+	+	+	+	+	+
*Oscillatoria* sp.	+	+	+	+	+	+	+	+
BACILLARIOPHYTA								
*Melosira* sp.	+	+		+	+			
*Coscinodiscus* sp.	+	+	+	+	+	+	+	+
*Stephanopyxis* sp.	+							
*Biddulphia* sp.		+	+		+	+	+	
*Pinnularia* sp.	+	+	+	+	+	+	+	+
*Navicula* sp.	+	+	+	+	+	+	+	+
*Gyrosigma* sp.	+	+	+	+		+		+
*Diploneis* sp.	+	+	+	+			+	+
*Diploneis* sp2.						+		+
*Plagiotropis* sp.	+	+	+	+	+	+	+	+
*Cocconeis* sp.	+	+	+	+	+	+	+	+
*Surirella* sp.	+	+	+					
*Gomphonema* sp.			+	+	+			
*Cymbella* sp.	+	+	+	+	+	+	+	+
*Licmophora* sp.	+	+	+	+	+	+	+	+
*Amphora* sp.		+	+	+	+	+		
*Cylindrotheca* sp.		+		+		+		+
*Nitzschia* sp.	+		+					
*Achnanthes* sp.		+	+	+		+	+	+
*Rhabdonema* sp.	+	+	+	+		+		
*Grammatophora* sp.	+	+	+	+	+	+	+	+
*Fragilaria* sp.	+	+	+	+		+		+
MIOZOA								
*Dinophysis* sp.						+		+
*Protoperidinium* sp.						+		
*Prorocentrum* sp.						+		+
CHLOROPHYTA								
*Spongomorpha pacifica*	+	+	+	+	+	+	+	+
*Ulothrix* sp.	+		+	+				
*Ulva clathrata*	+							
*Ulva lactuca*	+		+				+	+
*Ulva prolifera*	+		+					
*Ulva* sp.	+		+		+			+
*Rhizoclonium* sp.	+	+	+					
OCHROPHYTA								
*Ectocarpus siliculosus*	+	+	+	+				+
*Caepidium antarcticum*		+		+				
*Adenocystis utricularis*		+	+	+		+	+	+
*Scytosiphon lomentaria*		+		+		+		+
*Halopteris funnicularis*		+		+	+	+	+	+
*Macrocystis pyrifera*			+					
RHODOPHYTA								
*Acrochaetium* sp.	+			+	+	+		+
*Nothogenia fastigiata*	+	+	+	+	+	+	+	+
*Iridaea chordata*		+		+				+
*Sarcothalia crispata*						+		+
*Mazzaella laminaroides*								+
*Grateloupia* sp.		+		+				
*Ceramium* sp.		+			+			
*Heterosiphonia* sp.				+				
*Polysiphonia* sp.		+	+		+	+	+	+
*Pterosiphonia* sp.			+			+		
*Ballia callitricha*					+		+	
*Bostrychia* sp.			+		+	+	+	+
*Plocamium* sp.		+			+			
FORAMINIFERA								
Foraminifera indet	+	+	+	+	+	+	+	
MOLLUSCA								
*Margarella violacea*	+		+					
*Laevilitorina caliginosa*	+		+	+				
*Eatoniella* sp.	+							
*Onchidella marginata*				+				
*Mytilus platensis*	+	+	+	+		+	+	+
*Lasaea* sp.		+		+		+		
ARTHROPODA								
Amphipoda indet		+						
Ostracoda indet		+	+	+	+	+	+	
*Notochthamalus scabrosus*				+				
*Elminius kingii*						+	+	+
Arachnida indet	+	+	+	+				
*Halirytus magellanicus*	+	+	+	+				+

**Figure 2.**
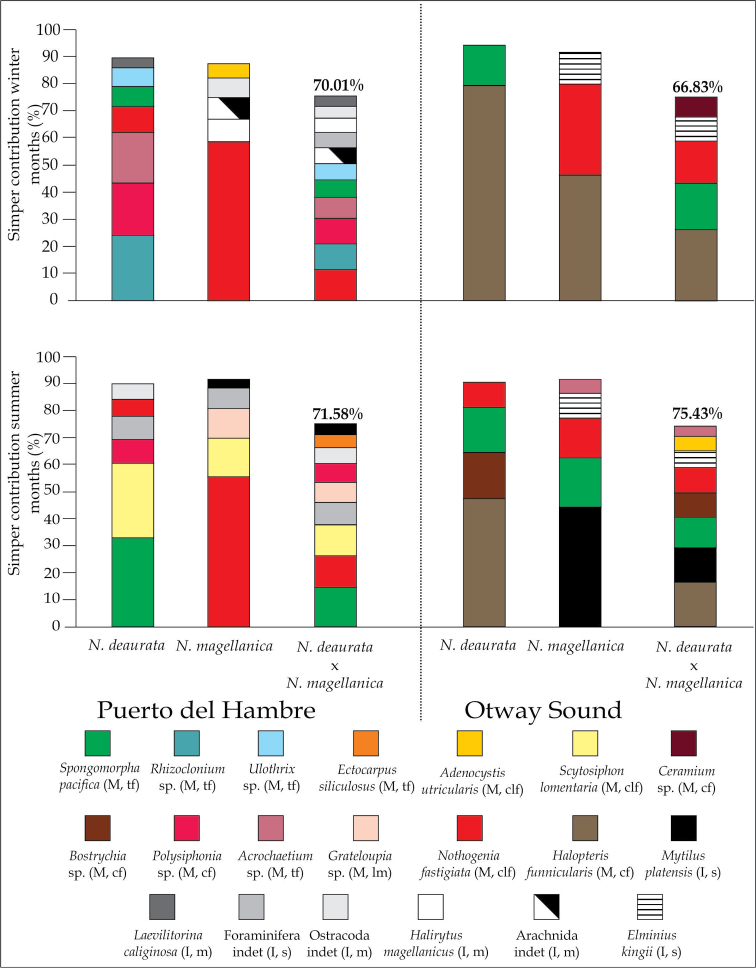
Percentage contribution SIMPER of items in the gut contents of the *Nacella* species in Puerto del Hambre and Otway Sound for the winter and summer months. The contribution limit was 90% of the total dietary composition. SIMPER analysis shows the dissimilarity between the species of *Nacella* in the two localities (average dissimilarity in bold and on the bar). The contribution limit was 75% of the total dietary composition. M = Macroalgae (with colours) and I = Invertebrates (with grey scale). Structural hardness of the thallus for macroalgae: th = thin filaments, cf = corticated filaments, clf = cylinder-like form and lm= leathery macrophyte. Functional group for invertebrates: s = sessile and m = mobile.

The macroscopic gut contents of both species of *Nacella* were characterized by 39 taxa, including green, brown and red macroalgae, as well as invertebrates such as foraminifera, molluscs and arthropods (Table 1). *Nacella deaurata* contained the highest total richness with 33 taxa, and *N. magellanica* had the highest occurrence frecuency of invertebrates (57 %). At temporal level, when comparing the dietary composition of the two species within each season separately (e.g., winter 1 × winter 2, winter 2 × winter 3 and summer 1 × summer 2 x, summer 2 × summer 3), there was no significant differences (PERMANOVA, *p* > 0.05) (Suppl. material 1: Tables S10, S11). However, significant differences (PERMANOVA, *p* < 0.05) (Suppl. material 1: Table S11) were observed in the dietary composition of both species between the winter and summer months for both species (e.g., winter 1 × summer 1), mainly due to the incorporation of different taxa (e.g., *S. pacifica*, *S. lomentaria*, *Bostrychia* sp., *M. platensis*) (Fig. 2) in the gut content during summer. In addition, these species did not present the same gut content composition, with significant differences between localities (PERMANOVA, *p* = 0.0001) (Suppl. material 1: Table S11) and mean dissimilarities over 50% (SIMPER). Specifically in the *N. deaurata* diet, the macroalgae *H. funnicularis*, *Bostrychia* sp., *Rizoclonium* sp., *S. lomentaria*, *S. pacifica* and *Polysiphonia* sp. contributed significantly to the dissimilarity between the two localities (SIMPER) (Fig. 2). The macroalgae *H. funnicularis*, *S. lomentaria*, *Grateloupia* sp., *N. fastigiata* and the invertebrates *M. platensis*, *E. kingii*, Archnida indet. and *H. magellanicus* contributed significantly to the dissimilarity of the diet of *N. magellanica* between localities (Fig. 2).

**Figure 3.**
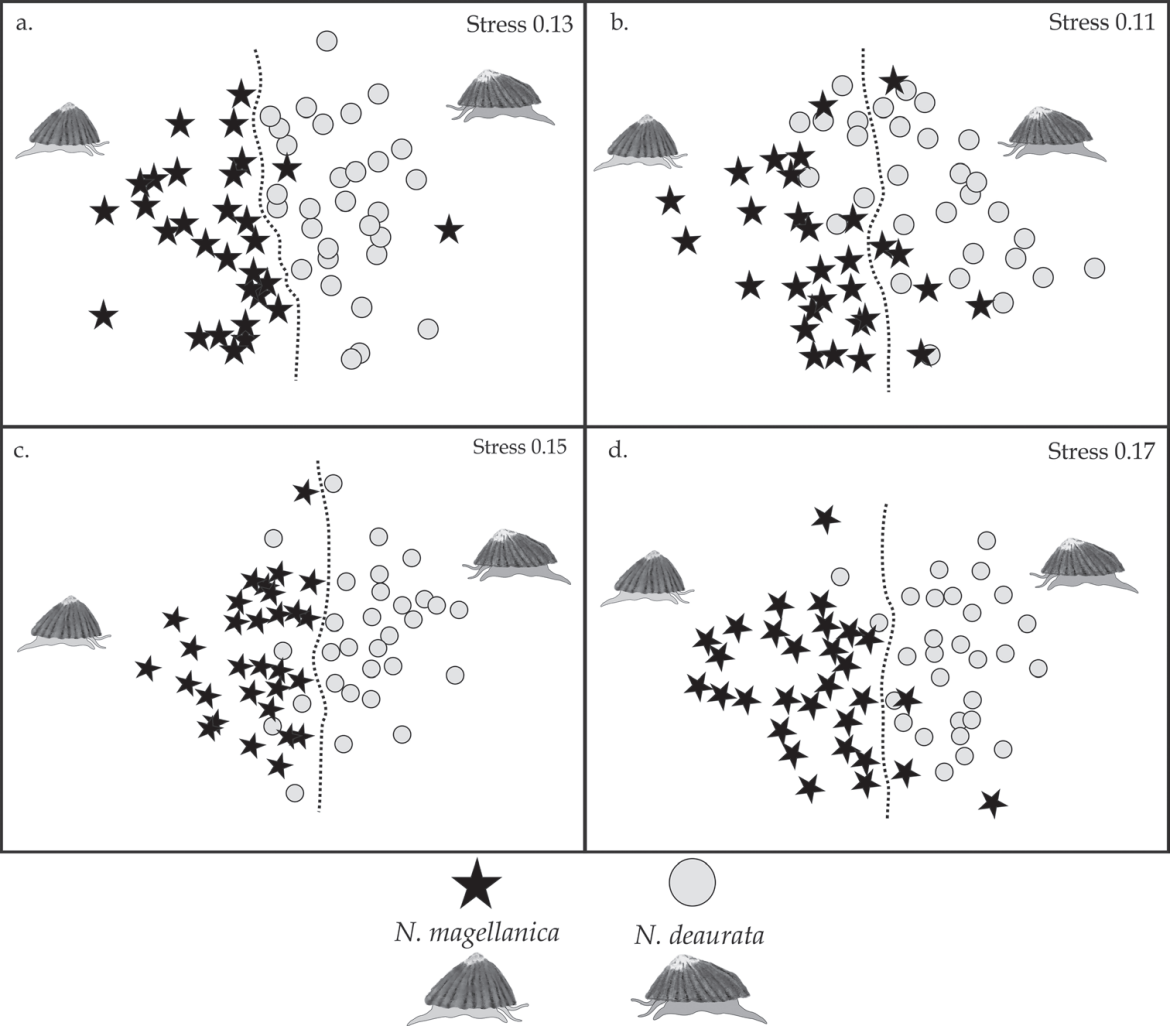
Non-metric multidimensional scaling of the dietary composition recorded in the gut contents of the *Nacella* species in Puerto del Hambre (**a, c**) and Otway Sound (**b, d**). **a, b** correspond to the winter months, and **c, d** to the summer months. The dashed line indicates the separation between species.

Finally, the dietary composition varied significantly between *N. deaurata* and *N. magellanica*, at each localities and each time (See PERMANOVA, *p* < 0.05, Suppl. material 1: Table S11 and NMDS, Fig. 3), and high dissimilarity averages were observed (> 65 %); SIMPER; Fig. 2). The diet of *N. deaurata* was generally composed of macroalgae taxa such as *H. funnicularis*, *S. pacifica*, *Bostrychia* sp., *Rizoclonium* sp., *Polysiphonia* sp., *Acrochaetium* sp., *N. fastigiata*, and *Ultothrix* sp. contributed to 90 % of similarity of the group. However, in the diet of *N. magellanica*, we observed a greater number of contributing invertebrates, such as *M. platensis*, *H. magellanicus*, *E. kingii*, Foraminifera indet. and Arachnida indet. (Fig. 2).

## Discussion

This is the first study to perform a temporal and spatial detailed analysis of the diet of intertidal *Nacella* species in the Sub-antarctic Ecoregion of Magellan. In general, both localities had a temporal and spatial variation in the composition of periphyton and macroalgae. In terms of diet, our results showed that in the gut contents of *N. deaurata* and *N. magellanica* we found a great variety of periphyton, macroalgae and some invertebrates. The results also demonstrated a temporal and spatial influence effect in the diet composition of both species. The diet composition between the two species was also different, mainly due to the highest occurrence of invertebrates in the gut content of *N. magellanica* and the highest occurrence of corticated filamentous macroalgae in *N. deaurata*. Here, we discuss how the high temporal and spatial variability of the benthic communities of periphyton and macroalgae affect in the dietary composition of two common grazers that inhabit the Magellan coast.

### Temporal and spatial variation in micro- and macroalgae assemblages

In general, the average dry biomass of the macroalgae assemblage, per quadrat, at each locality showed a significant increase during the summer months, with the greatest richness and abundance found at the middle intertidal level (Suppl. material 1: Table S4, S5). The multivariate analysis showed differences in the assemblage composition of micro and macroalgae between the two localities at each intertidal level and time. This dynamics of macroalgae assemblages in the channels and fjords of the Magellan Ecoregion was also observed by Ojeda (2013), who observed that the macroalgae assemblages do not present a general spatial structuring pattern, and there may even be differences in the structure of macroalgae assemblages between sites for similar intertidal levels within a same Bay (Ojeda 2013). This author also found that the macroalgae assemblages temporally presented a decrease in wet biomass in winter periods and a considerable increase in summer, showing the marked temporal dynamics of the macroalgae assemblages. These results suggest indetectable general pattern in the vertical zonation of micro- and macroalgae assemblages in these two localities (this study). In the channels and fjords of the Magellan Ecoregion, it has been described that the local environmental heterogeneity likely plays a role in structuring ecological assemblages and communities in Sub-Antarctic marine channels (Ríos et al. 2007, Aldea et al. 2011, Ojeda 2013, Ojeda et al. 2014). Finally, our data suggest an absence of a vertical pattern zonation between the two sites.

### Periphyton in the gut contents of *N. deaurata* and *N. magellanica*

**Figure 4.**
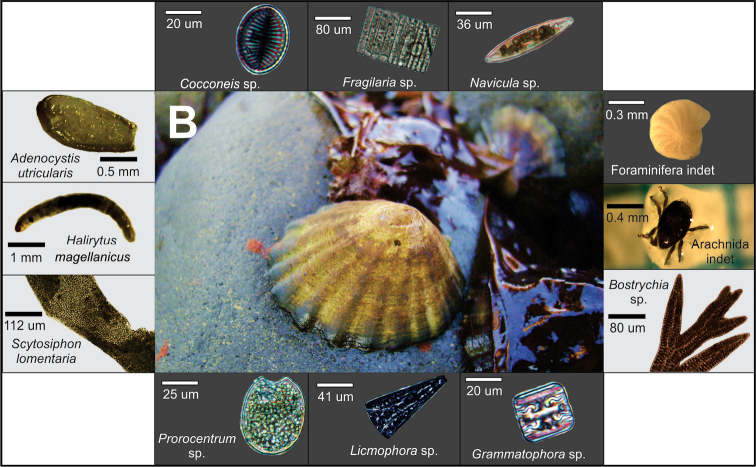
Light microscope and stereomicroscope images of microalgae, macroalgae and invertebrates taken from gut contents of *Nacella magellanica*.

In this study, the gut content of both species of *Nacella* presented a great variety of periphyton taxa (27 items), among which the most common were 12 taxa of diatoms and the cyanophyte *Chroococcus*. The rarest items were the dinoflagellates *Alexandrium*, *Dinophysis* and *Protoperidinium*, whose habits are mainly planktonic (Reguera et al. 2014), suggesting that their consumption was probably accidental. However, dinoflagellates have already been reported in the gastric contents of *Patella* species (Rubio et al. 2015). In addition, the dinoflagellate *Prorocentrum* sp. was recorded in summer at the two study locations; but in this case, its consumption cannot be considered accidental, since this dinoflagellate has a benthic habit (Tapia et al. 2002) (Fig. 4). In the present study, *Prorocentrum* sp. was also recorded in the quadrat samples in both localities. The record of this dinoflagellate is very important, mainly because it is associated with diarrheic toxins (DSP; Tapia et al. 2002), and might impact local people who consumes *Nacella* species (Ojeda 2013), even if they are not an official fishery in the Magellanic Region. Consequently, further study of *Prorocentrum* sp. dynamics would be crucial to determine whether this benthic periphyton is commonly found in grazer gastropods, especially *Nacella* and *Fissurella* limpets, who are locally consumed.

The abundance and composition of benthic diatoms can vary significantly between different micro-habitats within the rocky shore (Hill and Hawkin 1991, Jenkins et al. 2001). Similar dynamics of the periphyton community have been observed in the present study, since significant differences in the microalgae composition between different intertidal /zonation levels and betwenn, the two localities were observed. Hence, the differences observed for microalgae composition between both *Nacella* species could be associated with its vertical distribution.

Both *Nacella* species presented a shift in dietary composition in microalgae between the winter and summer months. A similar pattern was observed from the quadrats sampling (habitat composition). This shift in dietary composition is related to the temporal dynamics of periphyton communities, mainly due to the incorporation of different microalgae in the diet during summer. In the Northern Hemisphere, the seasonality in the composition of benthic microalgae has already been observed (Attard et al. 2014), and the greatest diversity and abundance were reached during the winter season (Jenkins et al. 2001). Also, studies in high latitudes of the Northern Hemisphere in *Patella vulgata* have shown that this species presents a seasonal change in the dietary composition mainly associated with changes in microalgae composition and abundance during the year (Hill and Hawkin 1991). In this study, the greatest diversity of microalgae was reached during summer; this is probably due to the higher productivity of benthic communities during summer in the Magellan coast (Ojeda 2013).

Analyses of gut contents showed greater richness and relative abundance than those observed in the quadrats. Studies in *P. vulgata* have shown similar results, with a greater variety of diatoms being found in the gut contents (Hill and Hawkin 1991). The authors explained that this is mainly due to these patellogastropods covering greater areas for longer times, and are much more effective when extracting periphyton from rock. The ability of herbivorous molluscs to extract microalgae directly depends on their radular structure (Steneck and Watling 1982, Ocaña and Fa 2003). For example, *Siphonaria* species have a radular structure composed of numerous small non-mineralized teeth, which are better suited for scraping soft foliate macroalgae than benthic diatoms (Ocaña and Fa 2003). On the other hand, the patellogastropod radula is a complex structure with mineralized teeth containing iron and silica, which are able to bore rock and remove microalgae from the substrate (Branch 1981). This feature allows them to highly consume periphyton, such as diatoms, algae spores, detritus and some invertebrates (Branch 1981, Safriel and Erez 1987). From a nutritional point of view, benthic microalgae are very important in the diet of many marine organisms, as they contain a high content of polyunsaturated fatty acids (PUFA) and eicosapentaenoic acid (EPA; Renaud et al. 1999, Vicose et al. 2012). In this sense, *Nacella* species, like other patellogastropods in the world (Branch 1981), present significant consumption of periphyton in the winter and summer seasons. Considering the important reduction of macroalgae cover and biomass during the Magellanic winter (Ojeda 2013, this study), the communities of periphyton constitute an important resource for *Nacella* species.

### Temporal and spatial variation in dietary composition of macroalgae and invertebrates

Variations in the diet of herbivores are generally correlated to food availability (Aguilera 2011). In our study, between winter and summer, both *Nacella* species shifted their dietary composition. In general, for the winter months in both species and in the two localities, the macroalgae that presented the highest relative abundances in the gut contents corresponded to the filamentous group (thin + corticated) (e.g *S. pacifica*, *H. funnicularis*, *Polysiphonia* sp.). Similarly, in both locations, the filamentous macroalgae contributed between a 33 (Puerto del Hambre) and a 51 % (Otway Sound) of the total biomass of the assemblages (Table 2). During the summer months, the filamentous macroalgae represented between a 7 (Puerto del Hambre) and a 28 % (Seno Otway) of the total biomass, while the cylinder-like form macroalgae (e.g. *A. utricularis*, *N. fastigiata*) increased their abundance, representing between 18 and 40 % of the total biomass of the assemblages. This temporal change in the assemblage was reflected in the dietary composition of both *Nacella* species, since in both localities, an increased in the relative abundance of cylinder-like form macroalgae in the gut contents were observed (Table 2).Therefore, the temporal variability of the macroalgae abundance was reflected in the diet, thus suggesting a generalist habit for these species. The temporal variation in dietary composition of herbivores also depends strongly on the latitude at which they live. For example, in subtropical rocky coasts, the dietary composition of *Megathura crenulata* does not vary during the year (Villarreal et al. 2013). In the lower latitudes of the Northern Hemisphere, the gut of *Turbo brunneus* contains a lot of corallinaceous algae all year without temporal differences (Ramesh and Ravichandran 2008). However, towards higher latitudes, seasonal climatic changes are stronger, and the results differ according to the marked seasonality of micro- and macroalgae assemblages on the coast (Jenkins et al. 2001, Ojeda 2013, this study). *Patella vulgata*’s diet varies with the composition and abundance of microalgae and filamentous macroalgae (Hill and Hawkin 1991). Seasonal variations in the diet of Chilean herbivorous molluscs have also been described, thus implying a change in food availability between the seasons (Aguilera 2011).

**Table 2.** Average dry biomass (g) of the different functional groups of macroalgae for the winter and summer months in the locality of Puerto del Hambre and Otway Sound and average relative abundance (%) of the different functional groups of macroalgal found in the gut contents of the two *Nacella* species. The values correspond to means ± DS.

Puerto del Hambre	Macroalgae (g)		*N. deaurata* (%)		*N. magellanica* (%)	
	winter	summer	winter	summer	winter	summer
Thin sheet-like forms	0.64±0.11	1.87±0.51	3±1	8.55±1.9	0.55±0.28	4.55±1.97
Thin filaments	2.51±1.19	3.95±1.01	11±2.3	21.55±4.6	3.22±1.4	4.55±2.28
Corticated filaments	1.09±0.3	1.67±0.50	6±2.6	5.11±1.44	2.33±1.3	2.77±2.43
Cylinder-like forms	1.96±0.65	8±2.36	1±0.3	5.44±1.5	5.88±2.56	9.11±1.57
Coenocytic forms	0.006±0.0001	0.12±0.12	0	0	0	0
Cushion-like forms	0.05±0.04	0	0	0	0	0
Leathery macrophyte	4.34±2.19	4.14±1.70	0	3.33±1.0	0.44±0.30	3.55±1-29
Otway Sound						
Thin sheet-like forms	2.14±0.66	1.69±0.58	0.22±0.15	0	0.56±0.56	1.22±0.49
Thin filaments	0.78±0.62	0.42±0.13	8±2	12.56±3.46	1.44±0.73	15.56±3.88
Corticated filaments	8.99±2.58	2.81±1.04	22.67±3.76	31±4.86	8±2.07	3.33±1.57
Cylinder-like forms	3.39±1.19	8.59±1.86	1.56±0.84	8.44±2.89	3.33±1.01	7.78±1.85
Coenocytic forms	0	0.29±0.29	0	0	0	0
Cushion-like forms	0	0.002±0.002	0	0	0	0
Leathery macrophyte	3.56±1.23	31.98±7.03	0	1.22±0.52	0	1.78±0.59

Generalist grazers use an opportunistic strategy in their feeding habits, consuming the most common resources available (Camus et al. 2012). Therefore, the significant differences observed in the dietary composition of *N. magellanica* and *N. deaurata* between the localities of Puerto del Hambre and Otway Sound reflect differences in macroalgae assemblages between sites, and highlight the generalist nature of these species. Similarly, differences in the diet of *M. crenulata*, along different rocky habitats of the west coast of the Baja California Peninsula (Villarreal et al. 2013) probably reflect changes in the benthic community among the localities. In the Magellanic Province, our results suggest that the marked environmental heterogeneity that leads to strong spatial variations in richness, abundance and structure of the benthic communities, plays a key role in the dietary variation of *Nacella* species (Ríos et al. 2007, Mansilla et al. 2013, Ojeda et al. 2014, this study).

Steneck and Watling (1982) classified herbivores molluscs according to their type of radula, and commented that the docoglossa radula of patellogastropods mainly excavates rock and extracts microalgae and filamentous algae, but does not consume leathery macrophytes. However, Raffaelli (1985) found that this pattern of classifying herbivores according to the type of radula may vary according to species characteristics and food availability. Among algal functional groups, the macroalgae with thin and corticated filaments as well as cylinder-like forms were preferentially consumed by *N. magellanica* and *N. deaurata*. Indeed, *N. deaurata* at both localities tends to consume more thin and corticated filamentous macroalga, as is the case in Otway Sound, despite the high abundance of leathery macrophyte species such as *Sarcothalia crispata*. Therefore, this greater tendency to consume more of a certain functional group of macroalgae could be related to the physiological capacity of the radula of these species (Steneck and Watling 1982).

### Interspecific variations in dietary composition

The diet composition in macroscopic items of both *Nacella* species was varied with a great variety of taxa (37 taxa). Among the most common items ingested by *N. deaurata* were nine species of macroalgae and some invertebrates such as *Laevilitorina caliginosa*, Ostracoda indet and Foraminifera indet, while *N. magellanica* consumed seven species of macroalgae and a large variety of invertebrates, including juveniles of *M. platensis*, larvae of the chironomids *Halirytus magellanicus*, Archnida indet, Ostracoda indet and Foraminifera indet. This is the first report of a nacellid species consuming a chironomid (Fig. 4).

**Figure 5.**
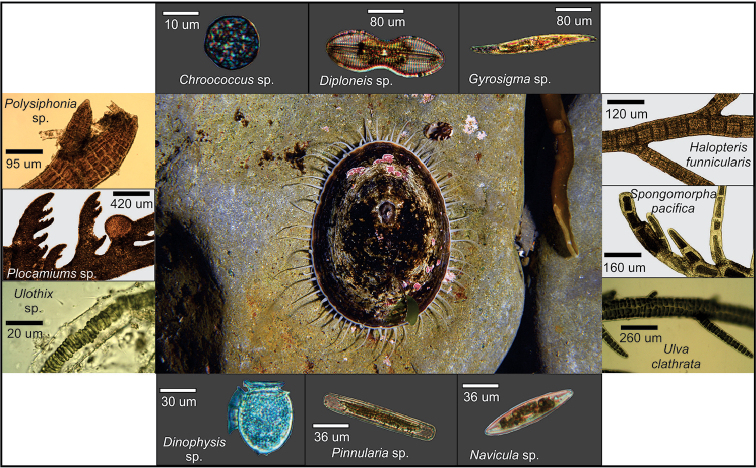
Light microscope and stereomicroscope images of microalgae, macroalgae and invertebrates taken from gut contents of *Nacella deaurata*.

Although *N. magellanica* and *N. deaurata* cohabit the intertidal zone, they differ markedly in their dietary composition, mainly because *N. deaurata* consumes a higher diversity of macroalgae (Fig. 5), whereas *N. magellanica* feed principally on invertebrates and macroalgae. Andrade and Brey (2014) reported similar results from the diets of 10 individuals of the same two species in Laredo Bay (Strait of Magellan). Their results, analyzing gut contents and stable isotopes, showed that *N. deaurata* behaves more herbivorous-like by consuming brown and red macroalgae, while *N. magellanica* consumes more invertebrates, and consequently is more omnivorous-like (by consuming green microalgae, micro-bivalves and foraminifera) (Andrade and Brey 2014). Therefore, this could suggest that the consumption of invertebrates by *N. magellanica* is not necessarily accidental. Recently in the Strait of Magellan, stable isotope analyses indicated that species of green and brown algae are preferentially consumed by macroherbivores, because they have higher nutritional value than red algae (Andrade et al. 2016). However, the consumption of some macroalgae species by generalist grazers such as *Nacella* species would more likely be linked to the algal availability in the habitat than to their nutritional profiles (Chapman and Underwood 1992). In this sense, it was observed that the *Nacella* species usually feed on common and abundant macroalgae such as the filamentous group (thin + corticated), which are fast growing and are available throughout the year (Ojeda 2013, this study) (Table 2), while other abundant groups of macroalgae, such as leathery macrophyte (e.g., *Sarcothalia crispata* or *Iridaea cordata*) did not show a high abundance in the gut contents (Table 2). In addition, recent nutritional studies in macroalgae from the Magellanic province (Astorga and Mansilla 2014) show that red algae such as *Pyropia*/*Porphyra* and *Callophylis* have higher energy and protein values than brown algae such as *Macrocystis pyrifera* and *Durvillaea antarctica*. In this study, two *Porphyra/Pyropia* morphotypes were reported for both localities, however no tissues were found in the gut contents of the *Nacella* species. Therefore, our results suggest that the consumption of groups of macroalgae species could be mainly related to the availability in the habitat and the structural hardness of the thallus. This has been observed for other marine gastropods. For instance, the specie *Turbo sarmaticus* mainly consumes *Ulva rigida* and *Gelidium pristoides* and, although *G. pristoides* presents higher nutritional values, it ingests mainly *U. rigida* because it has greater availability in the habitat than *G. pristoides* (Foster et al. 1999).

The differences observed in the dietary composition of *N. deaurata* and *N. magellanica* can also be explained by their vertical distribution in the coastal zone. For example, in the localities of Otway Sound and Puerto del Hambre, *N. magellanica* presented greater abundance in the middle zone compared to *N. deaurata*, while *N. deaurata* presented its highest abundances in the low intertidal zone and in the shallow subtidal (1 meter deep) (Rosenfeld 2016). In this sense, in Otway Sound, *N. magellanica* mainly consumes juveniles of *M. platensis*, which populations in the Magellanic Region settle commonly in the middle intertidal zone (Langley et al. 1980, Ojeda et al. 2014). In addition, we observed a marked difference in macroalgal assemblages between the vertical levels at both locations; indeed, the vertical zonation is crucial in the composition, richness, and biomass of macroalgae (Underwood 1980, Ojeda et al. 2014). Therefore, the vertical distribution of herbivores along the rocky shore can be determinant for their diet (Santina et al. 1993, Rubio et al. 2015). Indeed, a study on the dietary composition of the genus *Patella* showed that *P. rustica*, which occurs in the upper intertidal zone, only feeds on some species of algae, whereas *P. aspera* feeds on all types of algae (Santina et al. 1993). In Vancouver, the dietary composition of the species *Littorina sitkana* and *L. scutulata*, differs drastically mainly because *L. sitkana* is very rare in the upper intertidal zone, has low resistance to desiccation and a low capacity to collect food (Voltolina and Sacchi 1990). Finally, many of the differences in dietary composition between congeneric species depend on their zonal distribution on the shore. Consequently, the observed differences between *N. magellanica* and *N. deaurata* may be due to their vertical distribution, with *N. magellanica* being a more common inhabitant of the middle intertidal zone (Rosenfeld 2016).

In this study, how the variability in the composition of micro- and macroalgae among intertidal levels (middle and low) and between localities and time plays a fundamental role in the dietary composition of *N. magellanica* and *N. deaurata* was studied. This work is the first in the Magellanic Region to address the temporal and spatial characteristics of the diet of *Nacella* intertidal species, which provides a more complete listing of periphyton, macroalgae and invertebrates present in the diet of these Sub-Antarctic patellogastropods. Finally, it is important to mention that in this study only gut contents were analyzed. Therefore future research using new techniques such as stable isotopes could give a better resolution on the dietary composition that is effectively assimilated by *Nacella* species.

## Supplementary materials

Supplementary material 1 Tables S1–S11 Data type: Occurence of all algae taxa recorded, and Permutation analysis (PERMANOVA) of the composition of algae and gut content of Nacella species. Explanation note: This is a DOC file with all the temporal information of the occurrence of the algae taxa in both localities, and all the information of the PERMANOVA analyzes used in this study. This is an open access article distributed under the terms of the Creative Commons Attribution License (CC BY 4.0), which permits unrestricted use, distribution, and reproduction in any medium, provided the original author and source are credited.Sebastián Rosenfeld, Johanna Marambio, Jaime Ojeda, Juan Pablo Rodríguez, Claudio González-Wevar, Karin Gerard, Tamara Contador, Gemita Pizarro, Andrés Mansilla
