# Supplementary material for: Trophic ecology of two co-existing Sub-Antarctic limpets of the genus Nacella: spatio-temporal variation in food availability and diet composition of Nacella magellanica and N. deaurata
Source: Zookeys. 2018 Feb 19;(738):1–25. doi: 10.3897/zookeys.738.21175 (PMC5904503; doi:10.3897/zookeys.738.21175)
Supplement: Supplementary material 1 — Tables S1–S11 [file zookeys-738-001-s001.docx]

**Trophic ecology of two coexisting Sub-Antarctic limpets of the genus *Nacella*: Spatio-temporal variation in food availability and diet composition of *Nacella magellanica* and *N. deaurata* in the Sub-Antarctic Ecoregion of Magellan**

Sebastián Rosenfeld^1,2^ **·** Johanna Marambio^1,2^ **·** Jaime Ojeda^1^ **·** Juan Pablo Rodríguez^1,2^ **·** Claudio González-Wevar^1,2,3^ **·** Karin Gerard^1,3^ **·** Tamara Contador^4^ **·** Gemita Pizarro^5^ **·** Andrés Mansilla^1,2^

^1^Laboratorio de Macroalgas Antárticas y Subantárticas, Universidad de Magallanes, Casilla 113-D, Punta Arenas, Chile

^2^Instituto de Ecología y Biodiversidad (IEB) Casilla 653, Santiago, Chile

^3^GAIA Antártica – Universidad de Magallanes, Departamento de Recursos Naturales, Bulnes 01890, Punta Arenas, Chile.

^4^Parque Etnobotánico Omora, Universidad de Magallanes, Teniente Muñoz 396, Puerto Williams, Chile.

^5^Instituto de Fomento Pesquero, Casilla 101, Punta Arenas, Chile

Correspondence

Sebastián Rosenfeld Sekulovic, Laboratorio de Macroalgas Antárticas y Subantárticas, Universidad de Magallanes, Casilla 113-D, Punta Arenas, Chile. Instituto de Ecología y Biodiversidad (IEB) Casilla 653, Santiago, Chile. E-mail: [rosenfeld.sebastian@yahoo.com](mailto:rosenfeld.sebastian@yahoo.com)

**Table S1**.Systematic list of all algae taxa recorded, indicating their presence (+) in the winter and summer months, in Puerto del Hambre (P. Hambre) and Otway Sound (O. Sound).

|  |  | P. Hambre | | O. Sound | |
| --- | --- | --- | --- | --- | --- |
|  |  | Winter | Summer | Winter | Summer |
| **CYANOBACTERIA** | |  |  |  |  |
|  | *Chroococcus* sp. |  | + | + |  |
| **BACILLARIOPHYTA** | |  |  |  |  |
|  | *Achnanthes* sp. |  |  | + |  |
|  | *Cylindrotheca* sp. | + | + |  |  |
|  | *Coscinodiscus* sp. | + | + |  |  |
|  | *Cocconeis* sp. | + | + | + | + |
|  | *Cymbella* sp. | + | + |  | + |
|  | *Diploneis* sp. | + | + |  |  |
|  | *Diploneis* sp2. |  |  |  | + |
|  | *Fragilaria* sp. | + | + | + |  |
|  | *Grammatophora* sp. | + | + | + | + |
|  | *Licmophora* sp. | + | + | + | + |
|  | *Navicula* sp. | + | + | + | + |
|  | *Pinnularia* sp. | + | + | + | + |
|  | *Rhabdonema* sp. | + |  |  | + |
|  | *Surirella* sp. |  | + |  |  |
| **MIOZOA** | |  |  |  |  |
|  | *Dinophysis* sp. |  | + |  | + |
|  | *Prorocentrum lima* |  | + |  |  |
| **CHLOROPHYTA** | |  |  |  |  |
|  | *Bryopsis australis* |  |  |  | + |
|  | *Bryopsis* sp | + |  |  |  |
|  | *Chaetomorpha* sp | + |  |  |  |
|  | *Cladophora falklandica* |  | + |  | + |
|  | *Cladophora*sp | + |  |  |  |
|  | *Rhizoclonium tortuosum* |  | + |  |  |
|  | *Codium subantarcticum* | + | + |  |  |
|  | *Derbesia marina* |  | + |  |  |
|  | *Protomonostroma* sp | + |  |  |  |
|  | *Acrosiphonia arcta* |  |  |  | + |
|  | *Spongomorpha pacifica* | + | + | + | + |
|  | *Ulothrix* *implexa* | + | + |  |  |
|  | *Ulva clathrata* |  | + |  |  |
|  | *Ulva flexuosa* |  | + |  |  |
|  | *Ulva intestinalis* | + | + | + |  |
|  | *Ulva lactuca* | + | + | + | + |
|  | *Ulva torta* |  | + |  |  |
| **OCHROPHYTA** | |  |  |  |  |
|  | *Adenocystis utricularis* | + | + | + | + |
|  | *Caepidium antarcticum* |  | + |  |  |
|  | *Leathesia difformis* | + |  |  | + |
|  | *Cladostephus spongiosus* | + | + | + | + |
|  | *Durvillaea antarctica* |  |  | + | + |
|  | *Ectocarpus siliculosus* | + | + |  | + |
|  | *Petalonia fascia* | + | + |  |  |
|  | *Scytosiphon lomentaria* | + | + | + | + |
|  | *Scytothamnus fasciculatus* | + | + |  | + |
|  | *Halopteris funnicularis* | + |  | + | + |
| **RHODOPHYTA** | |  |  |  |  |
|  | *Acrochaetium* sp |  | + | + | + |
|  | *Ahnfeltia plicata* |  |  | + | + |
|  | *Ballia callitricha* | + | + | + | + |
|  | *Porphyra* sp1 | + | + |  | + |
|  | *Porphyra* sp2 |  | + | + | + |
|  | *Ptilonia magellanica* | + | + | + | + |
|  | *Bostrychia* sp |  |  | + | + |
|  | *Callithamnion gaudichaudii* | + | + | + | + |
|  | *Callithamnion montagnei* |  |  |  | + |
|  | *Ceramium diaphanum* |  | + | + |  |
|  | *Ceramium dozei* |  |  | + | + |
|  | *Ceramium pacificum* | + | + | + |  |
|  | *Ceramium stichidiosum* |  | + | + | + |
|  | *Ceramium virgatum* |  | + | + | + |
|  | *Acanthococcus antarcticus* |  |  | + | + |
|  | *Heterosiphonia berkeleyii* | + |  |  | + |
|  | *Heterosiphonia* sp1 | + | + |  | + |
|  | *Hymenena* sp |  |  | + |  |
|  | *Myriogramme* sp |  | + | + |  |
|  | *Schizoseris condensata* |  |  | + | + |
|  | *Chondria macrocarpa* |  |  | + | + |
|  | *Iridaea cordata* | + | + | + | + |
|  | *Mazzaella laminaroides* | + |  | + | + |
|  | *Sarcothalia crispata* |  | + | + | + |
|  | *Grateloupia* sp | + |  |  |  |
|  | *Phyllymenia* sp |  |  | + | + |
|  | *Ahnfeltiopsis furcellata* | + | + | + | + |
|  | *Plocamium* sp | + |  | + | + |
|  | *Lophurella hookeriana* | + | + | + | + |
|  | *Polysiphonia morrowii* | + | + | + | + |
|  | *Polysiphonia* sp1 |  | + |  |  |
|  | *Polysiphonia* sp2 |  | + |  | + |
|  | *Polysiphonia* sp3 |  |  | + |  |
|  | *Polysiphonia urceolata* |  | + |  |  |
|  | *Pterosiphonia* sp | + |  |  |  |

**Table S2**. Permutation analysis (PERMANOVA) of the composition of microalgae in Puerto del Hambre and Otway Sound. The design was of factorial type, considering localities, level and time. Data were based on Bray-Curtis dissimilarity and 9999 permutations were performed. Numbers in bold with asterisks indicate significant differences (p < 0.05).

| Source of variarion | df | SS | Pseudo-F | P |  |
| --- | --- | --- | --- | --- | --- |
|  |  |  |  |  |  |
| Localities (Lo) | 1 | 17297 | 7.888 | **0.0001*** |  |
| Level (Le) | 1 | 9154 | 4.175 | **0.0009*** |  |
| Time/Months (Ti) | 5 | 21848 | 1.993 | **0.0011*** |  |
| Lo x Le | 1 | 9786 | 4.463 | **0.0007*** |  |
| Lo x Ti | 5 | 77926 | 7.107 | **0.0001*** |  |
| Le x Ti | 5 | 14633 | 1.335 | 0.0873 |  |
| Lo x Le x Ti | 5 | 15061 | 1.374 | **0.0265*** |  |
| Res | 48 | 105250 |  |  |  |
| Total | 71 | 270960 |  |  |  |

**Table S3**. P-values for post-hoc comparisons conducted after PERMANOVA of composition of microalgae. Numbers in bold with asterisks indicate significant differences (p < 0.05).

| Localities | | Winter 1 | | Summer 1 | |
| --- | --- | --- | --- | --- | --- |
|  |  | Middle | Low | Middle | Low |
| P. Hambre x O. Sound | | **0.0383*** | **0.0195*** | **0.0128*** | **0.0221*** |
|  |  | Winter 2 | | Summer 2 | |
|  | | Middle | Low | Middle | Low |
| P. Hambre x O. Sound | | **0.0118*** | **0.0174*** | **0.0039*** | **0.002*** |
|  |  | Winter 2 | | Summer 2 | |
|  | | Middle | Low | Middle | Low |
| P. Hambre x O. Sound | | **0.0037*** | **0.0152*** | **0.006*** | **0.024*** |
| Level | | P. hambre | | O. Sound | |
|  |  | Winter 1 | Summer 1 | Winter 1 | Summer 1 |
| Middle x Low | | 0.4582 | **0.0089*** | 0.1570 | **0.0186*** |
|  |  |  |  |  |  |
|  | | Winter 2 | Summer 2 | Winter 2 | Summer 2 |
| Middle x Low | | 0.3972 | **0.0141*** | 0.2231 | **0.024*** |
|  |  |  |  |  |  |
|  | | Winter 3 | Summer 3 | Winter 3 | Summer 3 |
| Middle x Low | | 0.4832 | **0.0028*** | 0.2112 | **0.011*** |
| Time/Months | | P. hambre | | O. Sound | |
|  |  | Middle | Low | Middle | Low |
| Winter 1 x Winter 2 | | 0.2905 | 0.5619 | 0.5894 | 0.4975 |
| Winter 1 x Winter 3 | | 0.5592 | 0.6126 | 0.3674 | 0.5854 |
| Winter 1 x Summer 1 | | **0.0424*** | **0.0055*** | **0.0145*** | **0.015*** |
| Winter 1 x Summer 2 | | **0.0255*** | **0.0095*** | **0.0111*** | **0.024*** |
| Winter 1 x Summer 3 | | **0.0252*** | **0.0081*** | **0.0097*** | **0.0032*** |
| Winter 2 x Winter 3 | | 0.7011 | 0.8024 | 0.7031 | 0.9165 |
| Winter 2 x Summer 1 | | **0.0106*** | **0.0142*** | **0.025*** | **0.0282*** |
| Winter 2 x Summer 2 | | **0.0061*** | **0.0181*** | **0.0366*** | **0.0256*** |
| Winter 2 x Summer 3 | | **0.0052*** | **0.0189*** | **0.0207*** | **0.0165*** |
| Winter 3 x Summer 1 | | **0.0284*** | **0.0119*** | **0.0091*** | **0.0218*** |
| Winter 3 x Summer 2 | | **0.0155*** | **0.0141*** | 0.0778 | 0.0638 |
| Winter 3 x Summer 3 | | **0.0148*** | **0.012*** | 0.0768 | 0.0723 |
| Summer 1 x Summer 2 | | 0.5557 | 0.4355 | 0.881 | 0.7647 |
| Summer 1 x Summer 3 | | 0.8713 | 0.6775 | 0.8513 | 0.6212 |
| Summer 2 x Summer 3 | | 0.4558 | 0.9067 | 0.9893 | 0.7988 |

**Table S4**. Permutation analysis (PERMANOVA) of the richness (S) and abundance (N) of macroalgae in Puerto del Hambre and Otway Sound. The design was of factorial type, considering localities, level and time. Data were based on Euclidean distance. Numbers in bold with asterisks indicate significant differences (p < 0.05).

| Variable | Source of variarion | df | SS | Pseudo-F | P |
| --- | --- | --- | --- | --- | --- |
|  |  |  |  |  |  |
| S | Localities (Lo) | 1 | 0.002 | 0.017 | 0.8976 |
|  | Level (Le) | 1 | 4.384 | 38.370 | **0.0001*** |
|  | Time/Months (Ti) | 5 | 21.895 | 38.328 | **0.0001*** |
|  | Lo x Le | 1 | 0.370 | 3.238 | 0.0857 |
|  | Lo x Ti | 5 | 0.837 | 1.464 | 0.2163 |
|  | Le x Ti | 5 | 1.012 | 1.771 | 0.1409 |
|  | Lo x Le x Ti | 5 | 0.290 | 0.507 | 0.7678 |
|  | Res | 48 | 5.484 |  |  |
|  | Total | 71 | 34.273 |  |  |
|  | |  |  | |  |
| N | Localities (Lo) | 1 | 8.0012 | 19.73 | **0.0001*** |
|  | Level (Le) | 1 | 8.1621 | 20.1270 | **0.0001*** |
|  | Time/Months (Ti) | 5 | 49.909 | 24.614 | **0.0001*** |
|  | Lo x Le | 1 | 0.080 | 0.198 | 0.659 |
|  | Lo x Ti | 5 | 2.459 | 1.213 | 0.323 |
|  | Le x Ti | 5 | 3.150 | 1.554 | 0.193 |
|  | Lo x Le x Ti | 5 | 0.788 | 0.389 | 0.856 |
|  | Res | 48 | 19.465 |  |  |
|  | Total | 71 | 92.014 |  |  |

**Table S5.** P-values for post-hoc comparisons conducted after PERMANOVA of the richness (S) and abundance (N) of macroalgae in Puerto del Hambre and Otway Sound. Numbers in bold with asterisks indicate significant differences (p < 0.05).

| Level (S) | | P. hambre | | O. Sound | |
| --- | --- | --- | --- | --- | --- |
|  |  | Winter 1 | Summer 1 | Winter 1 | Summer 1 |
| Middle x Low | | **0.0146*** | **0.0032*** | 0.5388 | 0.3084 |
|  |  |  |  |  |  |
|  | | Winter 2 | Summer 2 | Winter 2 | Summer 2 |
| Middle x Low | | 0.0584 | **0.0016*** | **0.047*** | **0.0061*** |
|  |  |  |  |  |  |
|  | | Winter 3 | Summer 3 | Winter 3 | Summer 3 |
| Middle x Low | | 0.7232 | **0.0474*** | 0.2544 | 0.5359 |
| Time/Months (S) | | P. hambre | | O. Sound | |
|  |  | Middle | Low | Middle | Low |
| Winter 1 x Winter 2 | | 0.1032 | 0.1002 | 0.0655 | 0.194 |
| Winter 1 x Winter 3 | | 0.0973 | 0.0985 | 0.0588 | 0.1 |
| Winter 1 x Summer 1 | | **0.0147*** | **0.0292*** | **0.0488*** | **0.0265*** |
| Winter 1 x Summer 2 | | **0.004*** | **0.002*** | **0.0381*** | **0.038*** |
| Winter 1 x Summer 3 | | **0.0015*** | **0.0007*** | **0.0298*** | **0.016*** |
| Winter 2 x Winter 3 | | 0.051 | 0.1977 | 0.0895 | 0.1027 |
| Winter 2 x Summer 1 | | 0.1489 | 0.0137 | 0.4896 | 0.804 |
| Winter 2 x Summer 2 | | **0.0063*** | 0.2834 | **0.0235*** | 0.1894 |
| Winter 2 x Summer 3 | | **0.0007*** | **0.0243*** | **0.0031*** | **0.0215*** |
| Winter 3 x Summer 1 | | **0.0113*** | **0.0034*** | 0.1865 | **0.0338*** |
| Winter 3 x Summer 2 | | 0.2488 | **0.0092*** | 0.3549 | **0.0365*** |
| Winter 3 x Summer 3 | | **0.0232*** | 0.8324 | **0.0428*** | 0.203 |
| Summer 1 x Summer 2 | | 0.0908 | 0.1069 | 0.119 | 0.2205 |
| Summer 1 x Summer 3 | | 0.0969 | 0.1052 | 0.0588 | 0.1048 |
| Summer 2 x Summer 3 | | 0.1035 | 0.0991 | 0.1456 | 0.1033 |
| Localities (N) | | Winter 1 | | Summer 1 | |
|  |  | Middle | Low | Middle | Low |
| P. Hambre x O. Sound | | 0.5246 | 0.8594 | 0.1177 | 0.7942 |
|  |  | Winter 2 | | Summer 2 | |
|  | | Middle | Low | Middle | Low |
| P. Hambre x O. Sound | | **0.0224*** | **0.0106*** | **0.0015*** | **0.0137*** |
|  |  | Winter 2 | | Summer 2 | |
|  | | Middle | Low | Middle | Low |
| P. Hambre x O. Sound | | **0.0097*** | 0.1918 | **0.0041*** | **0.0403*** |
| Level (N) | | P. hambre | | O. Sound | |
|  |  | Winter 1 | Summer 1 | Winter 1 | Summer 1 |
| Middle x Low | | 0.2010 | 0.2674 | 0.5099 | 0.1206 |
|  |  |  |  |  |  |
|  | | Winter 2 | Summer 2 | Winter 2 | Summer 2 |
| Middle x Low | | 0.2069 | **0.0128*** | 0.7668 | **0.0068*** |
|  |  |  |  |  |  |
|  | | Winter 3 | Summer 3 | Winter 3 | Summer 3 |
| Middle x Low | | 0.5955 | **0.0044*** | 0.1457 | **0.0244*** |
| Time/Months (N) | | P. hambre | | O. Sound | |
|  |  | Middle | Low | Middle | Low |
| Winter 1 x Winter 2 | | 0.1014 | 0.0996 | 0.2357 | 0.0732 |
| Winter 1 x Winter 3 | | 0.0979 | 0.1002 | 0.1003 | 0.0563 |
| Winter 1 x Summer 1 | | **0.0017*** | 0.1359 | **0.0025*** | 0.4895 |
| Winter 1 x Summer 2 | | **0.0004*** | **0.0231*** | **0.012*** | **0.0437*** |
| Winter 1 x Summer 3 | | **0.0004*** | **0.0045*** | **0.0314*** | **0.0272*** |
| Winter 2 x Winter 3 | | 0.0972 | 0.099 | **0.0997** | 0.0858 |
| Winter 2 x Summer 1 | | **0.0124*** | 0.7317 | **0.025*** | 0.2443 |
| Winter 2 x Summer 2 | | **0.0006*** | 0.5319 | **0.0072*** | 0.0679 |
| Winter 2 x Summer 3 | | **0.001*** | 0.0517 | **0.0024*** | **0.0111*** |
| Winter 3 x Summer 1 | | 0.6723 | 0.263 | 0.3221 | 0.1395 |
| Winter 3 x Summer 2 | | **0.0102*** | **0.0361*** | **0.016*** | 0.9786 |
| Winter 3 x Summer 3 | | **0.0221*** | 0.5693 | **0.0023*** | 0.0842 |
| Summer 1 x Summer 2 | | 0.1702 | 0.9799 | 0.0969 | 0.1436 |
| Summer 1 x Summer 3 | | 0.0513 | 0.3958 | 0.1016 | 0.0688 |
| Summer 2 x Summer 3 | | 0.1225 | 0.1389 | 0.0992 | 0.0735 |

**Table S6**. Permutation analysis (PERMANOVA) of the composition of macroalge in Puerto del Hambre and Otway Sound. The design was of factorial type, considering localities, level and time. Data were based on Bray-Curtis dissimilarity and 9999 permutations were performed. Numbers in bold with asterisks indicate significant differences (p < 0.05).

| Source of variarion | df | SS | Pseudo-F | P |
| --- | --- | --- | --- | --- |
| Localities (Lo) | 1 | 40266 | 19.485 | **0.0001*** |
| Level (Le) | 1 | 14709 | 7.118 | **0.0001*** |
| Time/Months (Ti) | 5 | 21575 | 2.0881 | **0.0003*** |
| Lo x Le | 1 | 9943 | 4.8116 | **0.0002*** |
| Lo x Ti | 5 | 20659 | 1.9995 | **0.0002*** |
| Le x Ti | 5 | 12886 | 1.2472 | **0.0312*** |
| Lo x Le x Ti | 5 | 10775 | 1.0429 | **0.0022*** |
| Res | 46 | 95059 |  |  |
| Total | 69 | 227780 |  |  |

**Table S7.** P-values for post-hoc comparisons conducted after PERMANOVA of the composition of macroalgae in Puerto del Hambre and Otway Sound. Numbers in bold with asterisks indicate significant differences (p < 0.05).

| Localities | | Winter 1 | | Summer 1 | |
| --- | --- | --- | --- | --- | --- |
|  |  | Middle | Low | Middle | Low |
| P. Hambre x O. Sound | | **0.0002*** | **0.0118*** | **0.0008*** | **0.0127** |
|  |  | Winter 2 | | Summer 2 | |
|  | | Middle | Low | Middle | Low |
| P. Hambre x O. Sound | | **0.001*** | **0.0305** | **0.0078*** | **0.0071*** |
|  |  | Winter 2 | | Summer 2 | |
|  | | Middle | Low | Middle | Low |
| P. Hambre x O. Sound | | **0.004*** | **0.0026*** | **0.006*** | **0.0277*** |
| Level | | P. hambre | | O. Sound | |
|  |  | Winter 1 | Summer 1 | Winter 1 | Summer 1 |
| Middle x Low | | **0.015*** | **0.0009*** | **0.0159*** | **0.0012*** |
|  |  |  |  |  |  |
|  | | Winter 2 | Summer 2 | Winter 2 | Summer 2 |
| Middle x Low | | **0.0281*** | **0.0006*** | 0.0593 | **0.0008*** |
|  |  |  |  |  |  |
|  | | Winter 3 | Summer 3 | Winter 3 | Summer 3 |
| Middle x Low | | **0.0376*** | **0.0004*** | **0.039*** | **0.0001*** |
| Time/Months | | P. hambre | | O. Sound | |
|  |  | Middle | Low | Middle | Low |
| Winter 1 x Winter 2 | | 0.1107 | 0.2466 | 0.5894 | 0.4975 |
| Winter 1 x Winter 3 | | 0.2995 | 0.1983 | 0.3674 | 0.5854 |
| Winter 1 x Summer 1 | | **0.0001*** | **0.0263*** | **0.0001*** | **0.001*** |
| Winter 1 x Summer 2 | | **0.0001*** | **0.0001*** | **0.0001*** | **0.0084*** |
| Winter 1 x Summer 3 | | **0.0023*** | **0.0022*** | **0.0082*** | **0.0002*** |
| Winter 2 x Winter 3 | | 0.1004 | 0.8049 | 0.7031 | 0.9165 |
| Winter 2 x Summer 1 | | **0.0001*** | **0.0001*** | **0.0001*** | **0.0052*** |
| Winter 2 x Summer 2 | | **0.0001*** | 0.0778 | **0.0001*** | **0.0032*** |
| Winter 2 x Summer 3 | | **0.0001*** | **0.0001*** | **0.0001*** | **0.0015*** |
| Winter 3 x Summer 1 | | **0.0002*** | **0.0387*** | **0.0002*** | **0.0154*** |
| Winter 3 x Summer 2 | | **0.0001*** | 0.4973 | **0.0025*** | **0.002*** |
| Winter 3 x Summer 3 | | **0.0001*** | 0.6999 | **0.0058*** | **0.0078*** |
| Summer 1 x Summer 2 | | 0.4033 | 0.7007 | 0.881 | 0.7647 |
| Summer 1 x Summer 3 | | 0.5971 | 0.5957 | 0.8513 | 0.6212 |
| Summer 2 x Summer 3 | | 0.4065 | 0.5013 | 0.9893 | 0.7988 |

**Table S8.** Permutation analysis (PERMANOVA) of the composition of microalgae in the gut content of *Nacella* in Puerto del Hambre and Otway Sound. The design was of factorial type, considering localities, specie and time. Data were based on Bray-Curtis dissimilarity and 9999 permutations were performed. Numbers in bold with asterisks indicate significant differences (p < 0.05).

| Source of variarion | df | SS | Pseudo-*F* | *P* |
| --- | --- | --- | --- | --- |
|  |  |  |  |  |
|  |  |  |  |  |
| Localities (Lo) | 1 | 40191 | 69.176 | **0.0001*** |
| Specie (Sp) | 1 | 5447 | 9.3748 | **0.0001*** |
| Time/Months (Ti) | 5 | 36447 | 12.546 | **0.0001*** |
| Se x Lo | 1 | 6903 | 11.881 | **0.0001*** |
| Se x Sp | 5 | 19282 | 6.6374 | **0.0001*** |
| Lo x Sp | 5 | 11689 | 4.0236 | **0.0001*** |
| Se x Lo x Sp | 5 | 9835 | 3.3856 | **0.0001*** |
| Res | 216 | 125500 |  |  |
| Total | 239 | 255290 |  |  |

**Table S9.** P-values for post-hoc comparisons conducted after PERMANOVA of the composition of microalgae in the gut content of *Nacella* in Puerto del Hambre and Otway Sound. Numbers in bold with asterisks indicate significant differences (p < 0.05).

| Localities | | Winter 1 | | Summer 1 | |
| --- | --- | --- | --- | --- | --- |
|  |  | *N. deaurata* | *N. magellanica* | *N. deaurata* | *N. magellanica* |
| P. Hambre x O. Sound | | **0.0006*** | **0.0002*** | **0.0001*** | **0.0003*** |
|  |  | Winter 2 | | Summer 2 | |
|  | | *N. deaurata* | *N. magellanica* | *N. deaurata* | *N. magellanica* |
| P. Hambre x O. Sound | | **0.0001*** | **0.0001*** | **0.0001*** | **0.0013*** |
|  |  | Winter 3 | | Summer 3 | |
|  | | *N. deaurata* | *N. magellanica* | *N. deaurata* | *N. magellanica* |
| P. Hambre x O. Sound | | **0.0001*** | **0.0005*** | **0.0001*** | **0.0002*** |
| Species | | P. hambre | | O. Sound | |
|  |  | Winter 1 | Summer 1 | Winter 1 | Summer 1 |
| *N. deaurata* x *N. magellanica* | | 0.2575 | **0.0174*** | 0.5662 | **0.0001*** |
|  |  |  |  |  |  |
|  | | Winter 2 | Summer 2 | Winter 2 | Summer 2 |
| *N. deaurata* x *N. magellanica* | | **0.0006*** | **0.0001*** | **0.0212*** | **0.0001*** |
|  |  |  |  |  |  |
|  | | Winter 3 | Summer 3 | Winter 3 | Summer 3 |
| *N. deaurata* x *N. magellanica* | | **0.0007*** | **0.0003*** | **0.0312*** | **0.0005*** |
| Time/Months | | P. hambre | | O. Sound | |
|  |  | *N. deaurata* | *N. magellanica* | *N. deaurata* | *N. magellanica* |
| Winter 1 x Winter 2 | | 0.0638 | 0.0529 | 0.2648 | 0.0537 |
| Winter 1 x Winter 3 | | **0.0442*** | 0.0631 | 0.0551 | 0.22 |
| Winter 1 x Summer 1 | | **0.001*** | **0.0044*** | **0.0027*** | **0.0006*** |
| Winter 1 x Summer 2 | | **0.0001*** | **0.014*** | **0.0019*** | **0.0002*** |
| Winter 1 x Summer 3 | | **0.0002*** | **0.0017*** | **0.0008*** | **0.0001*** |
| Winter 2 x Winter 3 | | 0.2316 | 0.4072 | 0.2538 | 0.6885 |
| Winter 2 x Summer 1 | | **0.0002*** | **0.0003*** | **0.0001*** | **0.0003*** |
| Winter 2 x Summer 2 | | **0.0001*** | **0.0001*** | **0.0002*** | **0.0003*** |
| Winter 2 x Summer 3 | | **0.0001*** | **0.0014*** | **0.0001*** | **0.0001*** |
| Winter 3 x Summer 1 | | **0.0001*** | **0.0004*** | **0.0001*** | **0.0019*** |
| Winter 3 x Summer 2 | | **0.0001*** | **0.0001*** | **0.0006*** | **0.0005*** |
| Winter 3 x Summer 3 | | **0.0001*** | **0.0004*** | **0.0001*** | **0.0003*** |
| Summer 1 x Summer 2 | | 0.0702 | 0.199 | 0.3543 | 0.0797 |
| Summer 1 x Summer 3 | | **0.0413*** | **0.0344*** | 0.0569 | 0.0556 |
| Summer 2 x Summer 3 | | 0.1225 | 0.2172 | 0.0725 | 0.0928 |

**Table S10.** Analysis of permutations (PERMANOVA) of the composition of macroalgae and invertebrates in the gut content of *Nacella* in Puerto del Hambre and Otway Sound. The design was of factorial type, considering localities, specie and time. Data were based on Bray-Curtis dissimilarity and 9999 permutations were performed. Numbers in bold with asterisks indicate significant differences (p <0.05).

| Source of variarion | df | SS | Pseudo-F | *P* |
| --- | --- | --- | --- | --- |
|  |  |  |  |  |
|  |  |  |  |  |
| Localities (Lo) | 1 | 24102 | 9.6623 | **0.0001*** |
| Specie (Sp) | 1 | 41751 | 16.737 | **0.0001*** |
| Time/Months (Ti) | 5 | 45684 | 3.6628 | **0.0001*** |
| Se x Lo | 1 | 14509 | 5.8165 | **0.0001*** |
| Se x Sp | 5 | 75275 | 6.0353 | **0.0001*** |
| Lo x Sp | 5 | 34682 | 2.7807 | **0.0001*** |
| Se x Lo x Sp | 5 | 43277 | 3.4698 | **0.0001*** |
| Res | 206 | 513860 |  |  |
| Total | 229 | 801140 |  |  |

**Table S11.** P-values for post-hoc comparisons conducted after PERMANOVA of the composition of macroalgae and invertebrates in the gut content of *Nacella* in Puerto del Hambre and Otway Sound. Numbers in bold with asterisks indicate significant differences (p < 0.05).

| Localities | | Winter 1 | | Summer 1 | |
| --- | --- | --- | --- | --- | --- |
|  |  | *N. deaurata* | *N. magellanica* | *N. deaurata* | *N. magellanica* |
| P. Hambre x O. Sound | | **0.0007*** | **0.0002*** | **0.0001*** | **0.0001*** |
|  |  | Winter 2 | | Summer 2 | |
|  | | *N. deaurata* | *N. magellanica* | *N. deaurata* | *N. magellanica* |
| P. Hambre x O. Sound | | **0.0001*** | **0.0269*** | **0.0001*** | **0.0015*** |
|  |  | Winter 3 | | Summer 3 | |
|  | | *N. deaurata* | *N. magellanica* | *N. deaurata* | *N. magellanica* |
| P. Hambre x O. Sound | | **0.0005*** | 0.1351 | **0.0002*** | **0.0007*** |
| Species | | P. hambre | | O. Sound | |
|  |  | Winter 1 | Summer 1 | Winter 1 | Summer 1 |
| *N. deaurata* x *N. magellanica* | | 0.2230 | **0.0129*** | **0.0047*** | **0.0001*** |
|  |  |  |  |  |  |
|  | | Winter 2 | Summer 2 | Winter 2 | Summer 2 |
| *N. deaurata* x *N. magellanica* | | **0.0002*** | **0.0001*** | **0.0003*** | **0.0001*** |
|  |  |  |  |  |  |
|  | | Winter 3 | Summer 3 | Winter 3 | Summer 3 |
| *N. deaurata* x *N. magellanica* | | **0.0001*** | **0.0003*** | 0.0587 | **0.0005*** |
| Time/Months | | P. hambre | | O. Sound | |
|  |  | *N. deaurata* | *N. magellanica* | *N. deaurata* | *N. magellanica* |
| Winter 1 x Winter 2 | | 0.0548 | 0.1265 | 0.1975 | 0.1979 |
| Winter 1 x Winter 3 | | 0.6647 | 0.1758 | 0.2854 | 0.0625 |
| Winter 1 x Summer 1 | | **0.0178*** | **0.0003*** | **0.0001*** | **0.0001*** |
| Winter 1 x Summer 2 | | **0.0001*** | **0.0088*** | **0.0001*** | **0.0001*** |
| Winter 1 x Summer 3 | | **0.0046*** | **0.0309*** | **0.0001*** | **0.0001*** |
| Winter 2 x Winter 3 | | 0.2198 | 0.7836 | 0.5872 | 0.0577 |
| Winter 2 x Summer 1 | | **0.0001*** | **0.0002*** | **0.0001*** | **0.0012*** |
| Winter 2 x Summer 2 | | **0.0001*** | **0.0237*** | **0.0001*** | **0.0009*** |
| Winter 2 x Summer 3 | | **0.0001*** | **0.0171*** | **0.0001*** | **0.0079*** |
| Winter 3 x Summer 1 | | **0.0004*** | **0.0144*** | **0.0002*** | **0.0004*** |
| Winter 3 x Summer 2 | | **0.0001*** | **0.0005*** | **0.0003*** | **0.0005*** |
| Winter 3 x Summer 3 | | **0.0004*** | 0.1907 | **0.0001*** | **0.0007*** |
| Summer 1 x Summer 2 | | **0.0402*** | 0.1974 | **0.0381*** | 0.3319 |
| Summer 1 x Summer 3 | | 0.0519 | 0.0935 | 0.3463 | 0.2693 |
| Summer 2 x Summer 3 | | 0.4318 | **0.0387*** | 0.0693 | 0.1056 |
